# Hypolipidemic effect of ethanol extract from *Chimonanthus nitens* Oliv. leaves in hyperlipidemia rats via activation of the leptin/JAK2/STAT3 pathway

**DOI:** 10.1186/s10020-022-00589-z

**Published:** 2022-12-20

**Authors:** Jianping Pan, Xilin Ouyang, Qi Jin, Wei Wang, Jiali Xie, Baoming Yu, Zhijie Ling, Qizhen Wu, Baoping Zheng

**Affiliations:** 1 Department of Pharmacy, Gannan Healthcare Vocational College, Ganzhou, 341000 Jiangxi Province China; 2 College of Pharmacy, Gannan Medical University, Ganzhou, 341000 Jiangxi Province China; 3 Department of Physiology, Tongji Medical College, Huazhong University of Science and Technology, Wuhan, 430030 Hubei Province China; 4 Department of Basic Medicine, Gannan Healthcare Vocational College, Ganzhou, 341000 Jiangxi Province China; 5 Department of Clinical Medicine, Gannan Healthcare Vocational College, Ganzhou, 341000 Jiangxi Province China

**Keywords:** *Chimonanthus nitens* Oliv. leaf, Hyperlipidemia, Leptin/JAK2/STAT3 pathway, Lipid metabolism, Oxidative stress

## Abstract

**Background:**

This study aims to explore the protective role of ethanol extract from *Chimonanthus nitens* Oliv. leaf (COE) in hyperlipidemia via the leptin/Janus kinase 2 (JAK2)/signal transducer and activator of transcription 3 (STAT3) pathway.

**Methods:**

Male Sprague‒Dawley rats were randomly divided into 6 groups (n = 8): normal-fat diet (NMD), high-fat diet (HFD), HFD treated with simvastatin (SIM, 5 mg/kg/day), and HFD treated with COE (40, 80, 160 mg/kg/day). Lipid parameters, oxidative stress factors, serum leptin, body weight, hepatic wet weight and liver index were measured. Proteins in the leptin/JAK2/STAT3 pathway in liver tissues were determined using western blotting. Additionally, the expression levels of cytochrome P450 family 7 subfamily A member 1 (CYP7A1) and 3-hydroxy-3-methylglutaryl-CoA reductase (HMGCR) were quantified using western blotting and quantitative real-time polymerase chain reaction (qPCR).

**Results:**

COE decreased HFD-induced increases in body weight, hepatic wet weight and the liver index. HFD-induced hyperlipidemia and oxidative stress were observed in rat serum and livers. Additionally, COE repressed these two symptoms in rats fed a HFD. Moreover, COE caused CYP7A1 upregulation and HMGCR downregulation in HFD-fed rats. Mechanistically, COE induced the expression of leptin receptor (OB-Rb) and JAK2 and STAT3 phosphorylation in HFD-treated rats.

**Conclusion:**

COE activates the leptin/JAK2/STAT3 pathway, leading to an improvement in liver function and lipid metabolism and ultimately alleviating hyperlipidemia in rats. Therefore, COE may be a potential hypolipidemic drug for the treatment of hyperlipidemia.

## Highlights

1. COE improves lipid levels and lipase activities in hyperlipidemic rats.
2. COE ameliorates antioxidant enzyme activities and liver function in hyperlipidemic rats.
3. COE exerts a hypolipidemic effect by activating the leptin/JAK2/STAT3 pathway.

## Background

Hyperlipidemia is characterized by lipid metabolism dysfunction, with enhanced lipids or fats, and plays a role in the development and progression of cardiovascular and cerebrovascular diseases (Hill and Bordoni 2021; Oza et al. 2017). An eight-year study reported that the prevalence of hyperlipidemia increased in China from 2009 to 2016 (Gan et al. 2018). An investigation of 4105 individuals suggested that the prevalence of hyperlipidemia rose to 42.6% in Shanxi Province, China (Pan et al. 2018). Additionally, dyslipidemia crudely affected 35.8% of individuals aged ≥ 40 years (Xing et al. 2020) from 2017 to 2019 in China. Hyperlipidemia is one of the critical risk factors endangering health in China (Zhao et al. 2007). Effective treatment plays an important role in decreasing vascular disease and mortality, but it is limited by drug resistance, poor patient adherence, and adverse events that contribute to intolerance (Karr 2017). Novel therapeutic management is required to overcome the abovementioned weaknesses.

Chinese herbal medicine (CHM) has a protective role in hyperlipidemia, with a high curative effect and fewer side-effect events. CHM plays a potential role in controlling blood lipids and reducing the risk of cardiovascular events via excretory function enhancement, cardiovascular system improvement, and tonic effect reinforcement (Xie et al. 2012). *Chimonanthus nitens* Oliv. leaf, a typical CHM, is widely used for the treatment and management of colds and influenza (Huang et al. 2018). Mounting evidence suggests that ethanol extract of *Chimonanthus nitens* Oliv. leaf (COE) has application prospects in hyperlipidemia. A previous study showed anti-inflammatory effects of COE in zebrafish exposed to lipopolysaccharide (LPS) stimulation (Sun et al. 2017). Chen et al. concluded that COE ameliorated glycolipids and oxidative stress in a mouse model of type 2 diabetes mellitus (Chen et al. 2017). Additionally, a study based on a diabetic mouse model constructed by high-glucose and high-fat diet feeding and streptozotocin stimulation found that COE can promote glucose metabolism by increasing the expression of glucose transporter type 1 (GLUT1) and glucose transporter type 4 (GLUT4) (Chen et al. 2018). Thus, COE may be a potential drug for hyperlipidemia therapy due to its antioxidant and anti-inflammatory properties and its ability to regulate lipid metabolism. However, the clinical application of COE requires additional investigations that explore the concrete mechanism of CHM in disease progression.

The leptin/Janus kinase 2 (JAK2)/signal transducer and activator of transcription 3 (STAT3) pathway is significantly associated with the pathogenesis of hyperlipidemia via alterations in lipid metabolites. Leptin monitors the dynamic balance between energy intake and energy expenditure. Based on the interaction with leptin receptor (OB-R) leading to cellular pathway activation (Jequier 2002), leptin acts as the key regulatory factor in lipid metabolism (Hackl et al. 2019), autophagy (Cassano et al. 2014) and glucose absorption (El-Zein and Kreydiyyeh 2013) in the periphery. In the occurrence and development of hyperlipidemia, leptin production is negatively related to the level of triglycerides (TGs) (Lee et al. 2013). Leptin may contribute to hyperlipidemia through the OB-R-mediated activation of the JAK2/STAT3 pathway, which affects lipid metabolism and fat deposition in hepatic tissue (Wu et al. 2017; Gao et al. 2009; Chen et al. 2019). Cytochrome P450 family 7 subfamily A member 1 (CYP7A1) and 3-hydroxy-3-methylglutaryl-CoA reductase (HMGCR) are key antioxidant enzymes that show differential expression in hyperlipidemia (Xiao-Rong et al. 2021; Hu et al. 2021). Potentially, the leptin/JAK2/STAT3 pathway can modulate their expression to regulate disordered lipid metabolism. Thus, we hypothesize that CHM may be modulate this pathway and has potential application prospects in hyperlipidemia treatment.

Thus, we propose that the leptin/JAK2/STAT3 pathway may be the regulatory target of COE in hyperlipidemia treatment. This work focuses on the therapeutic value of COE and aims to demonstrate the potential mechanism of COE in the occurrence and development of hyperlipidemia.

## Methods

### Animals

Forty-eight male Sprague‒Dawley (SD) rats weighing 180–200 g (Vital River, Beijing, China) were placed in standard conditions with 60% humidity under a 12 h light/dark cycle at 25 °C. Rats were provided free access to food and water. The study protocol was reviewed and approved by the Ethics Committee for Biomedical Research of Gannan Medical University (approval number: 2020366). For hyperlipidemia modeling, 40 rats were continually fed a high-fat diet for 4 weeks and compared with 8 rats fed a basal diet during the same period (control group).

### Drug treatment

HFD rats were randomly divided into 5 groups (n = 8) as follows: HFD group, animals were treated daily with normal saline orally for 4 weeks; Simvastatin (SIM) group, animals were treated with SIM (Sigma‒Aldrich, USA) at a dose of 5 mg/kg/day by oral gavage for 4 weeks; COE groups (COE-L, M, H), animals were treated daily with COE at doses of 40, 80, or 160 mg/kg by oral gavage for 4 weeks as previously described (Chen et al. 2017). *Chimonanthus nitens* Oliv. leaves were collected from plants cultivated in Sanqing Mountain in Yushan County (Jiangxi, China) and dried at 40 °C overnight. The leaves were processed to powder for ethanol extraction (1/50, w/v), which was conducted according to the methods described in a previous study (Sun et al. 2017).

### Weight and liver index assays

We determined the body weight and hepatic wet weight of rats after the animals were intraperitoneally anesthetized with 1.5% pentobarbital sodium (0.2 mL/100 g) according to humanitarian principles. The liver index was calculated according to the following method: liver index = (hepatic wet weight × 100)/body weight.

### Haematoxylin and eosin (H&E) staining

H&E staining was performed as described before (Hu et al. 2021). In brief, isolated rat livers were fixed in 4% paraformaldehyde for more than 24 h. Then, tissues were dehydrated with a gradient with alcohol and embedded in paraffin. Samples were cut into 3-μmthick slices and stained with H&E. Finally, the samples were observed with an optical microscope and the quantification of lipid droplets were counted.

### Biochemical quantification

The hepatic tissues of rats were homogenized in Tris–HCl buffer (pH 7.4, Solarbio, Beijing, China). Using a Sigma 1–14 K centrifuge (Sigma, Germany), blood samples were collected from the abdominal aorta for a nest assay and subsequently centrifuged at 3,000 rpm for 15 min to obtain serum samples. The levels of aspartate aminotransferase (AST), alanine aminotransferase (ALT), malondialdehyde (MDA), glutathione (GSH), glutathione peroxidase (GSH-Px), superoxide dismutase (SOD), triglycerides (TGs), total cholesterol (TC), low-density lipoprotein cholesterol (LDL-C), fatty acid synthetase (FAS), lipase (LIPA) and high-density lipoprotein cholesterol (HDL-C) were measured using commercial detection kits and a microplate reader (Thermo, USA). Serum TC, TG, and LDL-C kits were purchased from Zhicheng Biological Technology Co., Ltd. (Shanghai, China). Liver TC, TG, and LDL-C kits and FAS and LIPA kits were obtained from Shanghai Sino Best Biological Technology Co., Ltd. (Shanghai, China). HDL-C, SOD, MDA, GSH-Px, GSH, AST, and ALT kits were obtained from Nanjing Jiancheng Bioengineering Institute (Jiangsu, China).

### Quantitative real-time polymerase chain reaction (qPCR)

Total RNA was extracted from hepatic tissues using TRIzol reagent (Life Technologies, CA, US). After the measurement of RNA concentration using an Evolution One ultraviolet visible spectrophotometer (840-341400, Thermo Scientific, USA), the RNA was reverse transcribed to first-strand cDNA using an iScript™ cDNA synthesis kit (Bio-Rad, CA, US). Then, an UltraSYBR Mixture kit (Thermo Scientific, USA) was used for qPCR analysis on an Applied Biosystems 7500 Fast Dx Real-Time PCR System (Applied Biosystems, USA) using CYP7A1 primers (forward: 5′-CTC TAA ATG CCC TGC AGA TGA-3′; reverse: 5′-GGC ACG GCT AAT GAT TCT CT-3′) and HMGCR primers (forward: 5′-CCT CCA TTG AGATCCGGAGG-3′; reverse: 5′-AAGTGTCACCGTTCCCACAA-3′) to obtain the value of Ct. We used GAPDH (forward primer: 5′-GCAAGTTCAACGGCACAG-3ʹ; reverse primer: 5′-GCCAGTAGACTCCACGACAT-3′) as the internal reference gene. The normalized CYP7A1 and HMGCR expression levels were calculated according to the formula 2^−ΔΔCt^.

### Western blotting

Protein samples from the supernatant of hepatic tissue were electrophoretically separated via SDS‒PAGE and then transferred to polyvinylidene fluoride membranes (Millipore, USA). After blocking with bovine serum albumin for 90 min at room temperature, the membranes were incubated overnight at 4 °C with the following antibodies: anti-CYP7A1 (bs-21430R, 1:1000, Bioss, Beijing, China), anti-HMGCR (ab174830, 1:5000, Abcam), anti-Leptin (bs-0409R, 1:1000, Bioss), anti-OB-Rb (ab5593, 1:2000, Abcam), anti-JAK2 (ab108596, 1:5000, Abcam), anti-phosphorylated (p)-JAK2 (ab32101, 1:5000, Abcam), anti-STAT3 (ab68153, 1:2000, Abcam), anti-p-STAT3 (ab76315, 1:1000, Abcam), and anti-GAPDH (ab181602, 1:10,000, Abcam). Subsequently, the membranes were incubated with HRP-conjugated goat anti-rabbit IgG secondary antibody (bs-40295G-HRP, 1:10,000, Bioss) at room temperature for 1 h. An ECL kit (Solarbio, Beijing, China) was used to visualize the protein bands. The blots were imaged using an X-ray imaging system (Bio-Rad, USA). GAPDH was used as the internal reference.

### Enzyme-linked immunosorbent assay (ELISA)

Leptin in the serum of rats was measured using a Rat Leptin ELISA kit (E-EL-R0582c, Elabscience, Wuhan, China). Samples and standards were added to a 96-well plate previously coated with anti-leptin antibody and incubated for 90 min at 37 °C, followed by incubation with anti-biotinylated leptin for 1 h at room temperature after the liquid in the plate was discarded. Subsequently, the samples were incubated with HRP-streptavidin solution for 30 min and then cultured with substrate solution for 15 min. Stop solution was added to the wells to change the color from blue to yellow before the color intensity was measured at 450 nm using an enzyme-labeled instrument (51119180ET, Thermo Fisher, USA).

### Statistical analysis

Experimental data are shown as the mean ± SD, followed by analysis using GraphPad Prism 8.0 and SPSS 22.0 (IBM, USA) software. Statistical analysis was performed using one-way analysis of variance (ANOVA) with a post hoc analysis. A p value of < 0.05 was considered statistically significant.

## Results

### Effects of COE on body weight, hepatic wet weight and liver index

**Fig. 1:**
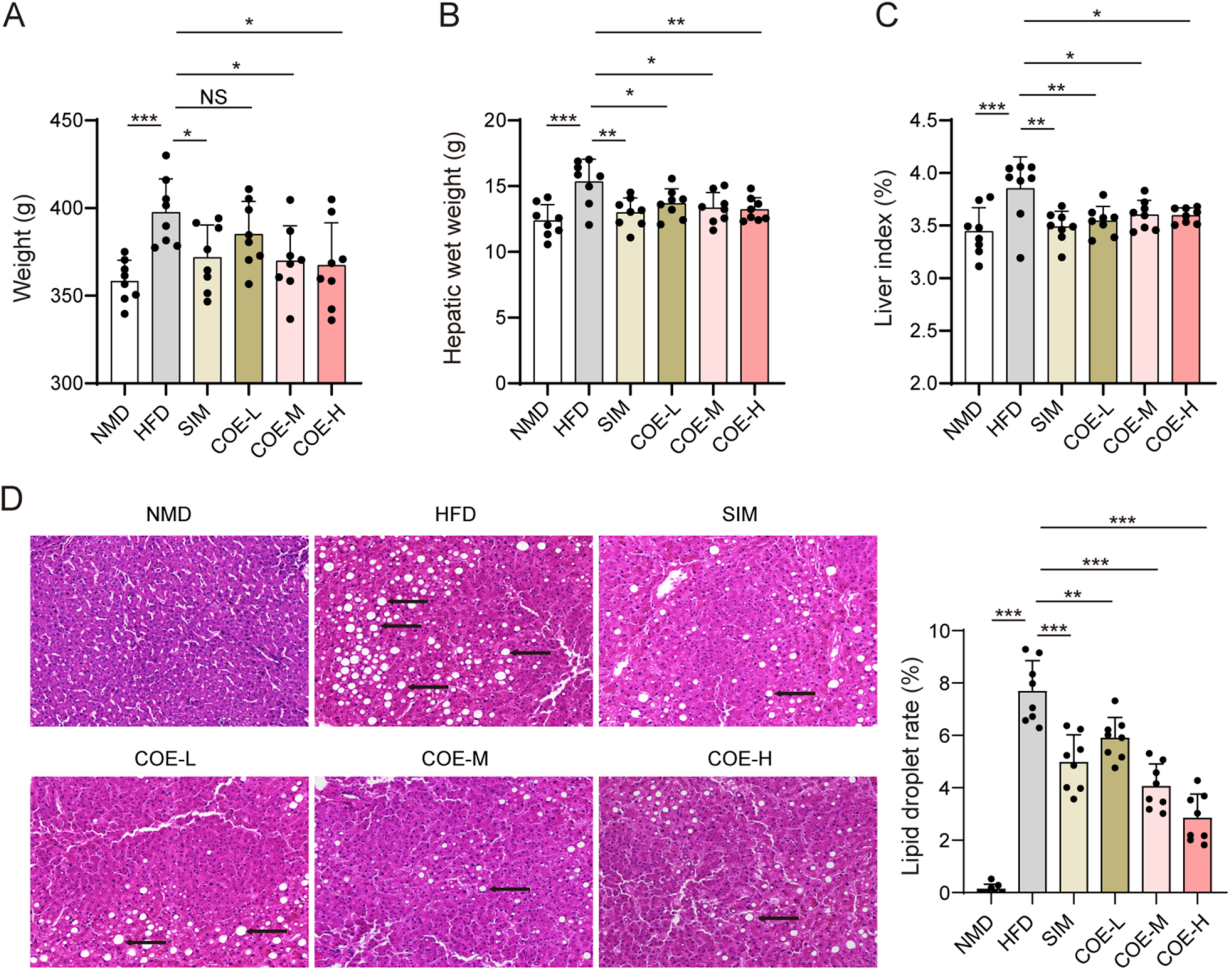
Effects of COE on body weight, hepatic wet weight and liver index in HFD-treated rats. **A** Body weight was measured at the end of the experimental period. **B** Hepatic wet weight was determined after COE or SIM treatment. **C** The liver index was assessed after COE or SIM treatment. **D** Histopathological changes in the liver of rats observed by H&E staining (black arrow indicated fat vacuole infiltration). NMD, normal diet. HFD, high-fat diet. SIM, simvastatin. COE, ethanol extract of *Chimonanthus nitens* Oliv. leaf. COE-L, COE at the low dose, COE-M, COE at the medium dose. COE-H, COE at the high dose. n = 8; *P < 0.05, **P < 0.01, ***P < 0.001 *vs*. NMD or HFD rats

To evaluate the effect of COE on the progression of hyperlipidemia, rats were randomly divided into 6 groups (NMD, HFD, SIM, COE-L, COE-M and COE-H), and the body characteristics of the rats were obtained, including body weight and hepatic wet weight. HFD feeding significantly increased the body weight and hepatic wet weight of rats (Fig. 1A, B). We calculated the liver index based on the two body characteristics of the rats. There was an elevation in the liver index in HFD-treated rats (Fig. 1C). Treatment with COE or SIM markedly decreased the three characteristics in HFD rats (Fig. 1A–C). HFD feeding remarkably increased lipid deposition; treatments with SIM and COE improved these histopathological changes in HFD rats (Fig. 1D). We concluded that COE can effectively improve the hepatic function of rats with hyperlipidemia based on the liver index.

### Effects of COE on lipid levels and lipase activities in hyperlipidemic rats

**Fig. 2:**
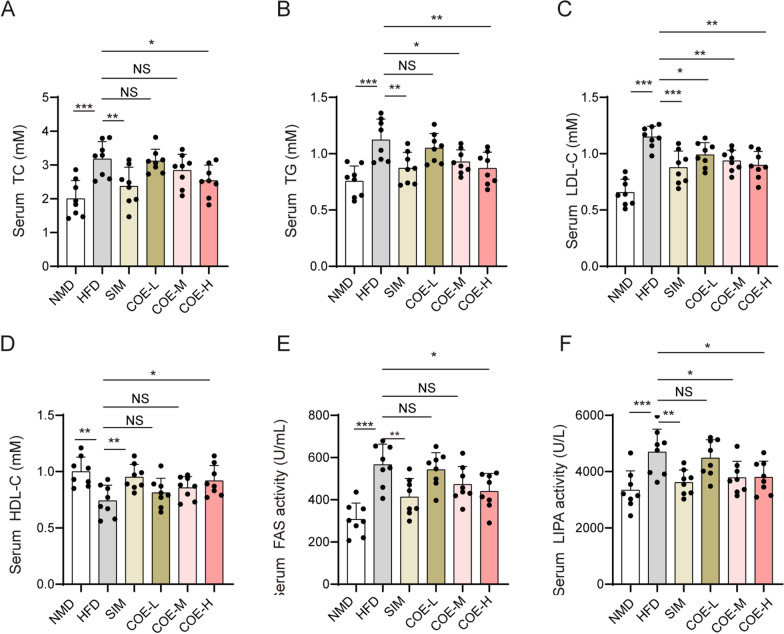
Effects of COE on lipid levels and lipase activities in the serum of hyperlipidemic rats. TC (**A**), TG (**B**), LDL-C (**C**), HDL-C (**D**), FAS (**E**), and LIPA (**F**) levels in the serum of hyperlipidemic rats were measured after COE or SIM treatment. n = 8; *P < 0.05, **P < 0.01, ***P < 0.001 *vs*. NMD or HFD rats

**Fig. 3:**
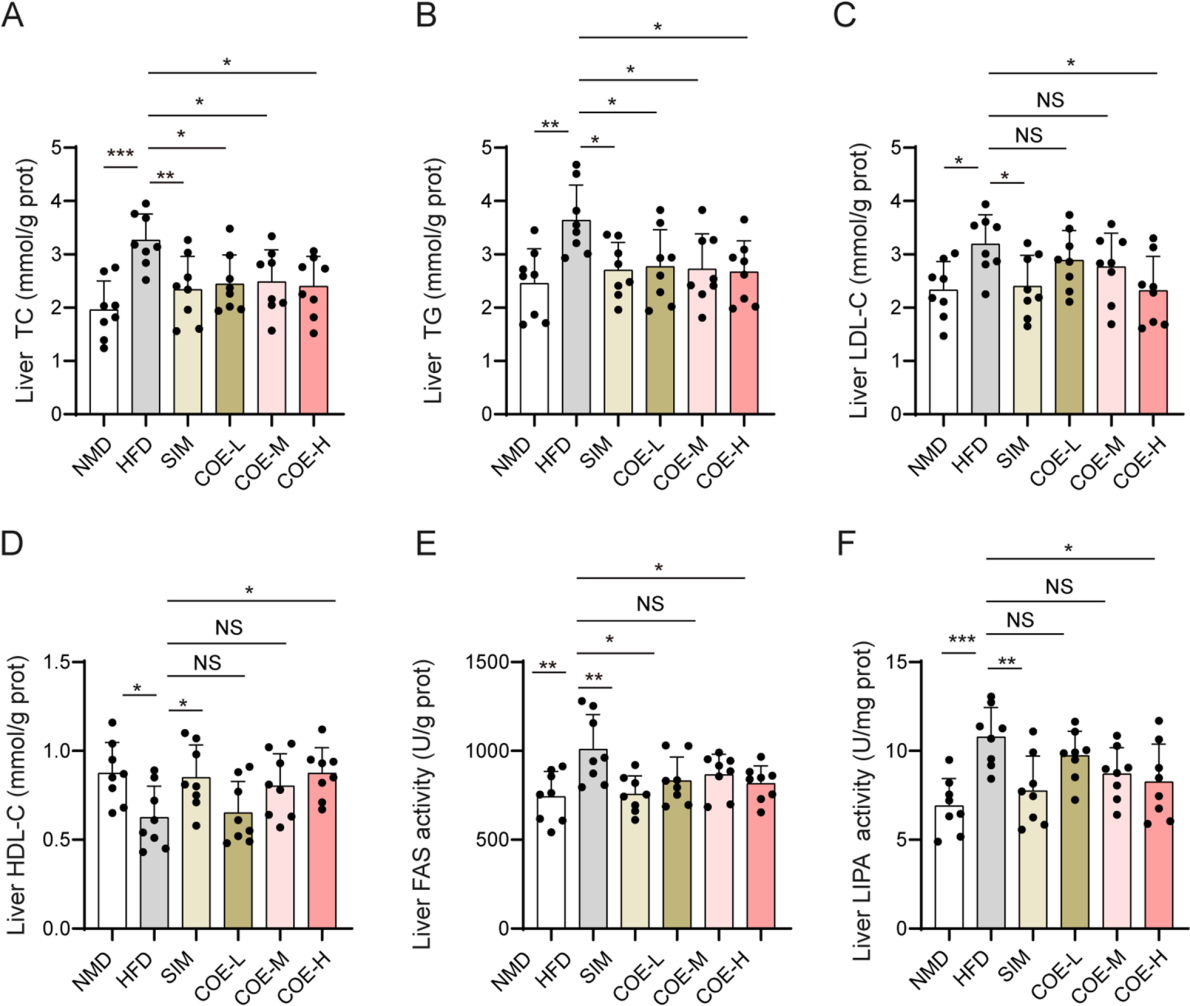
Effects of COE on lipid levels and lipase activities in liver tissues. TC (**A**), TG (**B**), LDL-C (**C**), HDL-C (**D**), FAS (**E**), and LIPA (**F**) levels in liver tissues of hyperlipidemic rats were measured after COE or SIM treatment. n = 8; *P < 0.05, **P < 0.01, ***P < 0.001 *vs*. NMD or HFD rats

We investigated the role of COE on the levels of lipids (TC, TG, LDL-C and HDL-C) and the activity of FAS or LIPA in hyperlipidemic rats. First, a HFD elevated TC, TG and LDL-C levels in the serum of rats (Fig. 2A–C) and caused an obvious drop in serum HDL-C levels (Fig. 2D). Moreover, serum FAS and LIPA were increased in HFD-induced rats (Fig. 2E, F). Similar to the effect of SIM, oral administration of COE, particularly at the high dose, resulted in significant declines in TC, TG, LDL-C, FAS and LIPA in the serum of HFD rats, with an enhancement of serum HDL-C (Fig. 2A–F). Next, changes in lipid levels and lipase activities in liver tissues were measured. The HFD enhanced TC, TG, LDL-C levels and FAS and LIPA activities while suppressing HDL-C levels in the liver tissues of rats (Fig. 3A–F). Treatment with SIM or COE markedly restored HFD-induced hepatic lipid levels and FAS and LIPA activities (Fig. 3A–F). These results indicate that COE significantly ameliorated lipid dysregulation and promoted lipase activities in hyperlipidemic rats.

### Effects of COE on antioxidant enzyme activities and liver function

**Fig. 4:**
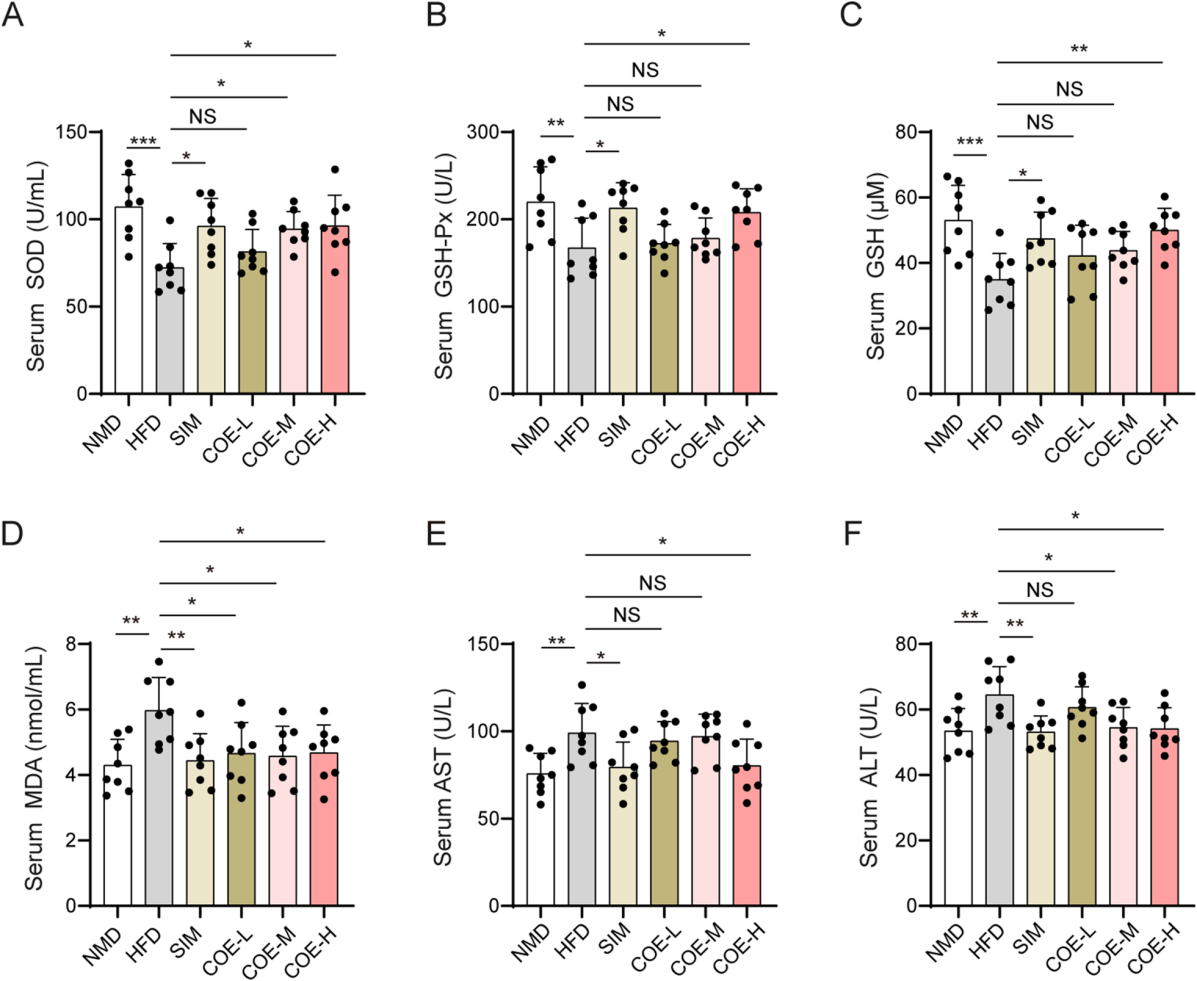
Effects of COE on serum antioxidant enzyme activities and liver function. SOD (**A**), GSH-Px (**B**), GSH (**C**), MDA (**D**), AST (**E**), and ALT (**F**) levels were examined in the serum of hyperlipidemic rats treated with COE or SIM. n = 8; *P < 0.05, **P < 0.01, ***P < 0.001 *vs*. NMD or HFD rats

**Fig. 5:**
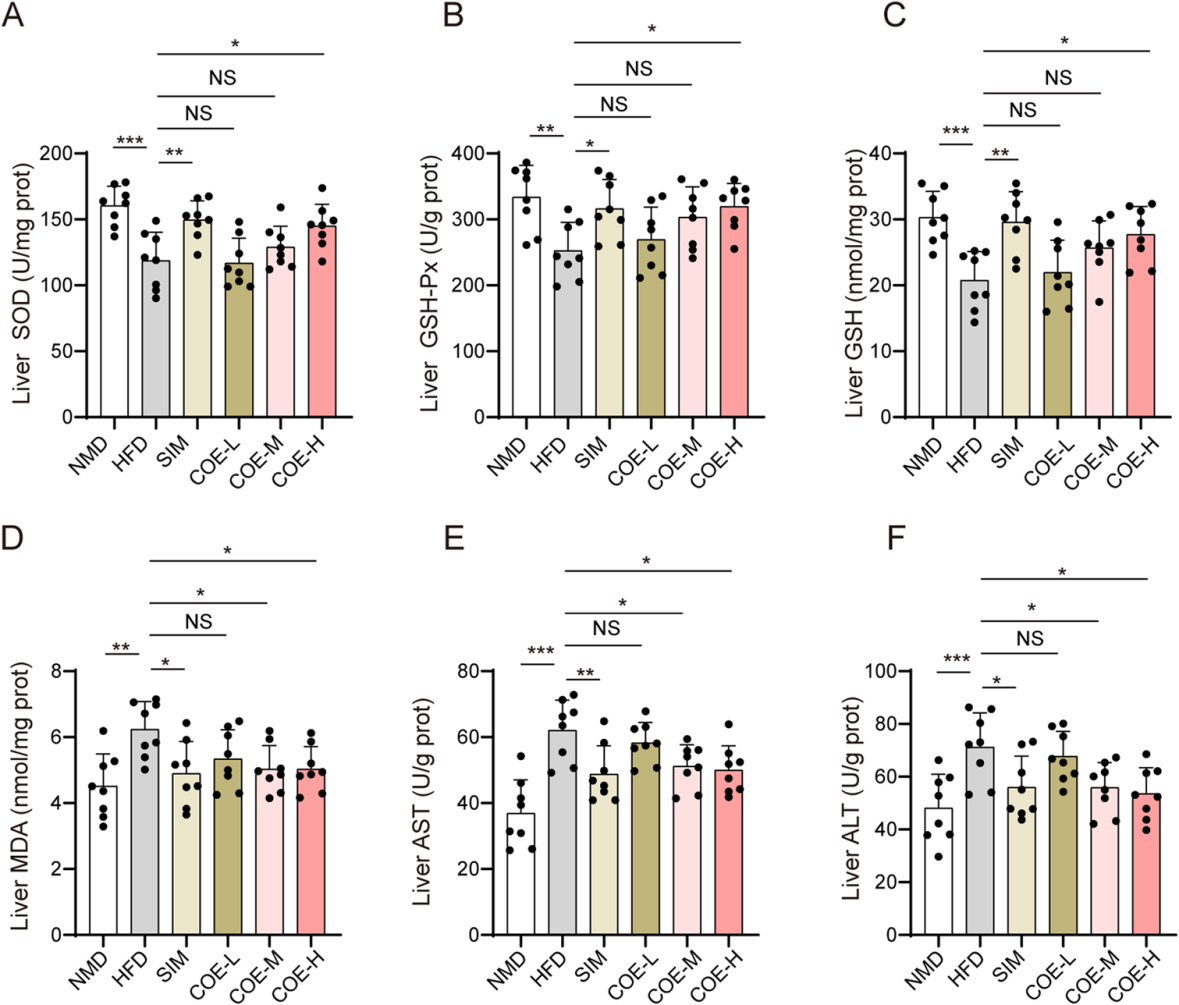
Effects of COE on antioxidase activities and liver function. SOD (**A**), GSH-Px (**B**), GSH (**C**), MDA (**D**), AST (**E**), and ALT (**F**) levels were examined in hepatic tissues of hyperlipidemia rats treated with COE or SIM. n = 8; *P < 0.05, **P < 0.01, ***P < 0.001 *vs*. NMD or HFD rats

Exposure to a HFD suppressed GSH production and the activities of SOD and GSH-Px in the serum of rats (Fig. 4A–C). Additionally, treatment with COE or SIM significantly enhanced HFD-suppressed SOD, GSH-Px and GSH levels in serum. Additionally, the HFD induced activation of AST or ALT and MDA production, whereas COE or SIM treatment reduced the HFD-induced MDA, AST and ALT enhancements in serum (Fig. 4D–F). In the hepatic tissue of rats, COE or SIM treatment significantly increased the HFD-decreased SOD, GSH-Px and GSH levels (Fig. 5A–C) and repressed the HDF-induced elevation in MDA, AST and ALT levels (Fig. 5D–F). These results suggest that COE can cause obvious improvements in oxidative stress and liver function in hyperlipidemic rats.

### Effects of COE on CYP7A1 and HMGCR expression

**Fig. 6:**
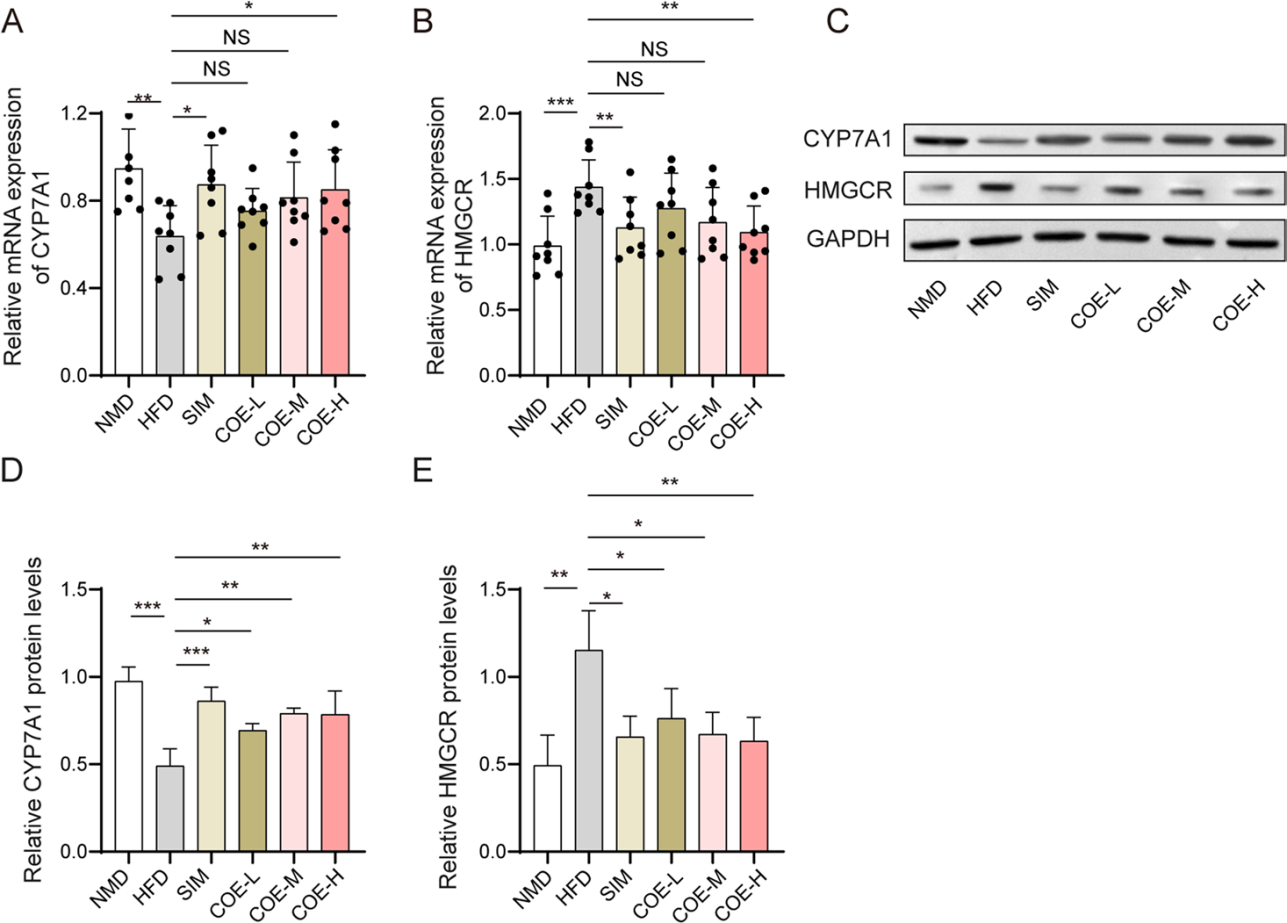
Effects of COE on CYP7A1 and HMGCR expression. **A** CYP7A1 mRNA levels were determined by qPCR. **B** HMGCR mRNA levels were measured via qPCR. **C**, **D** CYP7A1 and HMGCR protein levels were assessed through western blot assays. n = 8; *P < 0.05, **P < 0.01, ***P < 0.001 *vs*. NMD or HFD rats

As shown in Fig. 6A and B, COE or SIM treatment elevated the HFD-induced decrease in CYP7A1 mRNA levels (Fig. 6A) while reducing the HFD-induced increase in HMGCR mRNA expression (Fig. 6B). According to western blotting results, exposure to a HFD caused a decrease in CYP7A1 protein and an increase in HMGCR protein (Fig. 6C–E). Importantly, COE or SIM treatment significantly reversed these HFD-induced changes given the observed CYP7A1 enhancement and HMGCR reduction in HFD-treated rats (Fig. 6C–E). Collectively, these results indicate that COE might be involved in lipid metabolism by regulating the expression levels of CYP7A1 and HMGCR.

### Effects of COE on leptin/JAK2/STAT3 pathway activation in HFD-treated rats

**Fig. 7:**
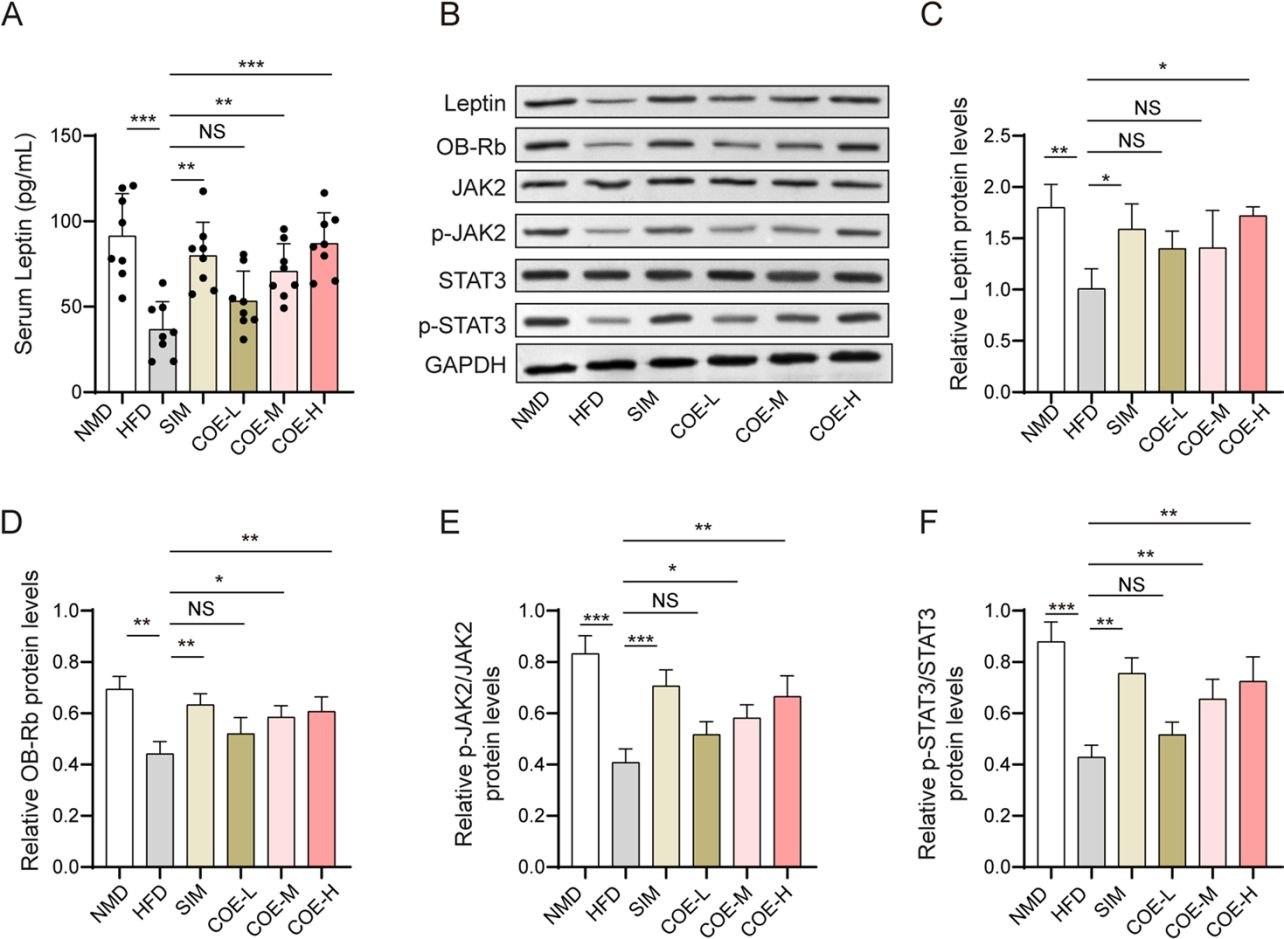
The role of COE in leptin/JAK2/STAT3 pathway activation. **A** Serum leptin levels were measured via ELISA. **B**–**F** Leptin and OB-Rb levels and JAK2 and STAT3 phosphorylation in hepatic tissues were examined via western blotting. n = 8; *P < 0.05, **P < 0.01, ***P < 0.001 *vs*. NMD or HFD rats

We examined whether COE would induce activation of the leptin/JAK2/STAT3 pathway in HFD-fed rats. In serum, COE increased the HFD-induced decrease in leptin levels (Fig. 7A). The HFD significantly reduced leptin and OB-Rb expression and the phosphorylation of JAK2 and STAT3 in liver tissues (Fig. 7B–F), which were markedly increased by treatment with COE or SIM (Fig. 7B–F). These results demonstrate that COE can activate the leptin/JAK2/STAT2 pathway in rats with hyperlipidemia.

## Discussion

The COE-diminished liver index observed in this study indicates that COE can ameliorate HFD-induced liver injury. Hyperlipidemia is characterized by a reduction in HDL-C and elevation of TC, TG and LDL-C (Das and Kumar 2021). In serum and liver samples from HFD rats, COE not only upregulated HFD-reduced HDL-C but also downregulated HFD-elevated TG, TC and LDL-C, with concomitant inactivation of FAS and LIPA. These results suggest that COE can inhibit lipid metabolism enzyme (FAS and LIPA) activities to restore the disordered lipid metabolism in hyperlipidemia (Evans et al. 2019; Jones and Infante 2015). Similarly, Chen et al. concluded that COE significantly suppressed the levels of TC, TG and LDL-C but enhanced the HDL-C level in diabetic mice with elevated lipids (Chen et al. 2017). Interestingly, we found that lipid metabolism-related enzymes, including CYP7A1 and HMGCR, were regulated by COE in HFD rats. CYP7A1 and HMGCR mediate the conversion from cholesterol to bile acid in the liver and thus have become potential drug targets to induce lipid-lowering effects in hyperlipidemia (Chambers et al. 2019; Xia et al. 2020; Istvan and Deisenhofer 2001). Therefore, COE may improve the elevated lipid phenotype via CYP7A1/HMGCR-mediated lipid metabolism.

Additionally, we showed that COE obviously enhanced the activities of antioxidant enzymes, inducing GSH upregulation and MDA downregulation, which are biomarkers of oxidative stress. COE has been demonstrated to possess an anti-inflammatory effect in LPS-stimulated RAW264.7 cells by decreasing the expression of TNF-α, IL-6 and IL-1β (Sun et al. 2017). Oxidative stress contributes to the occurrence and development of chronic inflammation in various chronic diseases (Hussain et al. 2016). An investigation of diabetic nephropathy rats suggested that a decrease in oxidative stress was inhibited following disorders in metabolism and organ function (Arora et al. 2019). In metabolic disorders, oxidative stress activates certain transcription factors that participate in the expression of inflammation-related genes, which ultimately causes inflammatory cytokines to be differentially expressed (Hussain et al. 2016). The inflammatory condition converts the metabolic phenotype from the resting state to the active state, thereby contributing to lipid metabolism dysfunction (McGarry et al. 2018). Thus, COE can interfere with the link between oxidative stress and inflammation to alleviate hyperlipidemia symptoms.

Leptin in the periphery reduces fat deposition, which contributes to hyperlipidemia improvement (Pereira et al. 2021). This anti-lipogenic effect depends on the interaction between leptin and OB-R, a leptin receptor belonging to the class I cytokine receptor family. OB-R isoforms are divided into 6 classes: OB-Ra, OB-Rb, OB-Rc, OB-Rd, OB-Re and OB-Rf (Gorska et al. 2010). We found that exposure to COE increased leptin expression and OB-Rb phosphorylation in HFD-fed rats, which indicates that the leptin/OB-Rb pathway mediates the protective role of COE in the elevated-lipid phenotype. Moreover, we showed the COE-induced activation of the JAK2/STAT3 pathway in the hepatic tissue of HFD-fed rats. OB-Rb includes intracellular motifs that can manipulate the activation of the JAK/STAT pathway (Fruhbeck 2006). COE shows the potential to modulate protein expression at the transcriptional and translational levels (Chen et al. 2018). Thus, we hypothesize that COE may elevate leptin expression to activate OB-Rb, which subsequently induces increased phosphorylation of the JAK2/STAT3 pathway involved in hepatic inflammation and lipid metabolism disorder (Chen et al. 2019).

Importantly, STAT3 phosphorylation is a potential upstream factor in the two mechanisms. A previous review concluded that the CYP7A1-involved decrease in bile acid was caused by STAT3 activation and hepatocyte nuclear factor 4α (HNF-4α) in the liver (Ibrahim et al. 2019). Geng et al. suggested that the JAK2/STAT3 pathway induced oxidative stress and inflammation in the myocardial tissue of HFD-fed rats (Geng et al. 2019). As described in a previous study based on liquid chromatography‒mass spectrometry, quercetin is the active component of COE (Chen et al. 2017). Importantly, quercetin can regulate the expression of leptin and OB-Rb to induce activation of the JAK2/STAT3 pathway (Chang et al. 2022). Thus, quercetin may mediate the role of COE in the leptin/OB-Rb/JAK2/STAT3 pathway in a rat model of hyperlipidemia. Collectively, the protective role of COE in hyperlipidemia is carried out via STAT3-involved oxidative stress and lipid metabolism.

## Conclusion

**Fig. 8:**
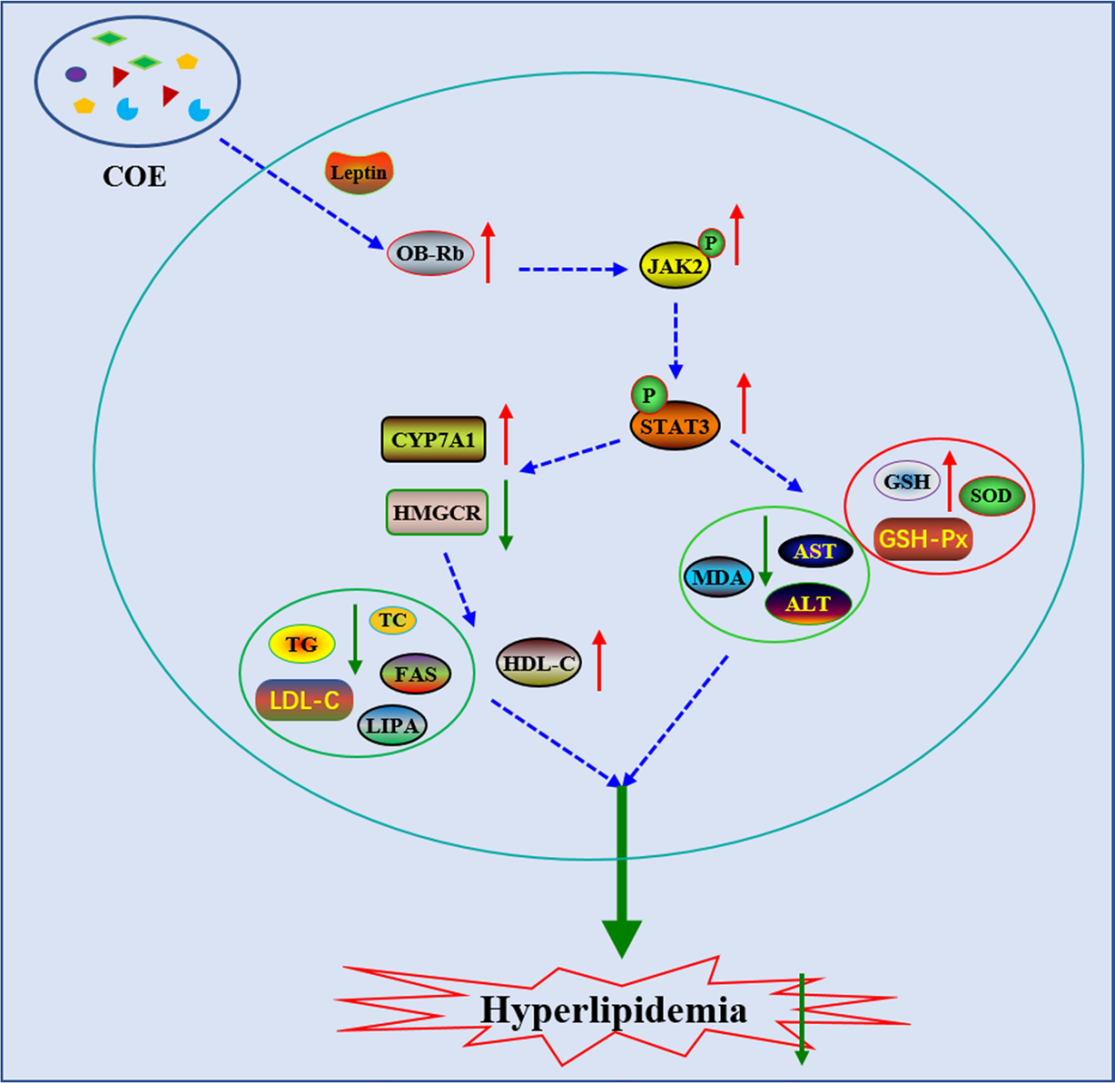
Potential novel mechanism of COE in hyperlipidemia treatment. COE promotes the interaction between leptin and OB-Rb, which induces phosphorylation of JAK2 and STAT3. On the one hand, the activation of the JAK2/STAT3 pathway elevates antioxidase activities and improves liver function. On the other hand, phosphorylation of STAT3 regulates lipid metabolism based on CAP7A1 elevation and HMGCR reduction, finally alleviating hyperlipidemia in rats. ↑: increase, ↓: decrease

We provide a novel mechanism of COE in hyperlipidemia treatment. COE increases OB-Rb-mediated phosphorylation of the JAK2/STAT3 pathway by promoting the interaction between leptin and OB-Rb, which leads to a) an improvement in hepatic function that is dependent on a decrease in oxidative stress and b) the recovery of lipid metabolism due to CYP7A1 enhancement and HMGCR reduction (Fig. 8).

## Data Availability

The datasets used or analyzed in the current study are available from the corresponding author on reasonable request.

## Abbreviations

COE: Ethanol extract of *Chimonanthus nitens* Oliv. leaf
COE-L: COE at the low dose
COE-M: COE at the medium dose
COE-H: COE at the high dose
HFD: High-fat diet
NS: No significance
OB-Rb: Leptin receptor
JAK2: Janus kinase 2
STAT3: Signal transducer and activator of transcription 3
AST: Aspartate aminotransferase
ALT: Alanine aminotransferase
MDA: Malondialdehyde
GSH: Glutathione
GSH-Px: Glutathione peroxidase
SOD: Superoxide dismutase
TG: Triglyceride
TC: Total cholesterol
FAS: Fatty acid synthetase
LIPA: Lipase
LDL-C: Low-density lipoprotein cholesterol
HDL-C: High-density lipoprotein cholesterol
CHM: Chinese herbal medicine
SIM: Simvastatin
CYP7A1: Cytochrome P450 family 7 subfamily A member 1
HMGCR: HMG-CoA reductase
